# Brain metastasis, *EGFR* mutation subtype and generation of EGFR-TKI jointly influence the treatment outcome of patient with *EGFR*-mutant NSCLC

**DOI:** 10.1038/s41598-023-45815-8

**Published:** 2023-11-21

**Authors:** Jia-Shiuan Ju, Allen Chung-Cheng Huang, Pi-Hung Tung, Chi-Hsien Huang, Tzu-Hsuan Chiu, Chin-Chou Wang, How-Wen Ko, Fu-Tsai Chung, Ping-Chih Hsu, Yueh-Fu Fang, Yi-Ke Guo, Chih-Hsi Scott Kuo, Cheng-Ta Yang

**Affiliations:** 1 Division of Thoracic Oncology, Department of Thoracic Medicine, College of Medicine, Chang Gung Memorial Hospital, Chang Gung University, Taoyuan City, Taiwan; 2 Thoracic Oncology Unit, Chang Gung Memorial Hospital Cancer Center, Taoyuan City, Taiwan; 3 Division of Pulmonary & Critical Care Medicine, Kaohsiung Chang Gung Memorial Hospital, Kaohsiung, Taiwan; 4 Data Science Institute, Department of Computing, Imperial College London, London, UK

**Keywords:** Cancer, Cancer therapy, Lung cancer, Metastasis

## Abstract

Non-small cell lung cancer (NSCLC) with epidermal growth factor receptor (EGFR) mutation is brain metastasis (BM)-prone. We determined the impact of this hallmark, along with *EGFR* subtype and generation of tyrosine kinase inhibitor (TKI) treatment, on patients’ outcome. 553 metastatic *EGFR*-mutant NSCLC patients received front-line EGFR-TKI treatment. Progression-free survival (PFS), overall survival (OS) and secondary T790M rate were analysed. BM was observed in 211 (38.2%) patients. BM (HR 1.20 [95% CI 0.99–1.48]; p = 0.053), ECOG PS 0–1 (HR 0.71 [95% CI 0.54–0.93]; p = 0.014) and afatinib treatment (HR 0.81 [95% CI 0.66–0.99]; p = 0.045) were associated with PFS. Afatinib-treated patients without BM demonstrated a significantly longer PFS (16.3 months) compared to afatinib-treated patients with BM (13.7 months) and to gefitinib/erlotinib-treated patients with (11.1 months) or without BM (14.2 months; p < 0.001). CNS-only progression trended higher in afatinib-treated patients. ECOG PS 0–1 (HR 0.41 [95% CI 0.31–0.56]; p < 0.001) and *EGFR* L858R mutation (HR 1.46 [95% CI 1.13–1.88]; p = 0.003), but not BM, were the predictors for OS. BM (OR 2.02 [95% CI 1.02–4.08]; p = 0.040), afatinib treatment (OR 0.26 [95% CI 0.12–0.50]; p < 0.001) and *EGFR* L858R mutation (OR 0.55 [95% CI 0.28–1.05]; p = 0.070) were associated with secondary T790M rate. In BM patients, gefitinib/erlotinib-treated ones with 19 deletion mutation and afatinib-treated ones with L858R mutation had the highest and the lowest T790M rate (94.4% vs. 27.3%, p < 0.001), respectively. BM and generation of EGFR-TKI jointly impact PFS and secondary T790M rate in patients with *EGFR*-mutant NSCLC, whereas OS was mainly associated with *EGFR* subtype.

## Introduction

Treatment of EGFR-TKI for advanced NSCLC patients with *EGFR*-sensitizing mutation is one of the supreme advances in lung cancer treatment today^1^. The first, second and third-generation EGFR-TKIs, with variable degree of therapeutic efficacies, are all currently standard of cares in this setting^2^. Although the survival outcome of *EGFR*-positive NSCLC patients has tremendously improved by these TKI treatments, the prognosis remains profoundly affected by certain clinical, molecular and therapeutic factors^3,4^.

Brain metastasis-prone nature is a hallmark of NSCLC with *EGFR* mutation; as earlier studies have revealed that *EGFR*-mutant patients demonstrated a higher rate of brain involvement compared to *EGFR*-wild type ones^5–8^. Presence of brain metastasis in *EGFR*-mutant patients may further impact the clinical outcome. This has been reported in front-line TKI-treated patients where, irrespective of first- or second-generation drugs administered, those with brain metastasis tended to have shorter PFS compared to those without^9–11^. Nevertheless, the impact of brain metastasis to OS has been relatively variable. Some studies have shown that it was associated with a worse OS in *EGFR*-positive patients^12,13^, partly due to a negative influence on patients’ performance status whereas some others have demonstrated that TKI-treated patients with brain metastasis experienced a similar OS as those without^14–16^, likely associated with the favorable intracranial efficacies of these drugs. Therefore, more clinical practice-based, brain metastasis-dedicated analyses from TKI-treated *EGFR*-mutant patients are warranted for elucidation.

In addition, generation of EGFR-TKI used also serves as an outcome-associated factor in *EGFR*-mutant patients. Notably, third-generation osimertinib treatment has exhibited a significantly longer PFS and OS compared to first-generation TKI treatment^17^. With good CNS penetration, osimertinib also significantly reduces BM not only in patients with advanced stage disease but also in those with early stage disease as an adjuvant treatment^17,18^. However, the wide use of front-line osimertinib remains unachievable in many countries due to the high cost. The second-generation afatinib has demonstrated superior efficacy over first-generation TKI in the LuxLung-7 trial^19^, whereas the finding did not appear to be consistently observed in real-world settings^11,20,21^. Dacomitinib, another second-generation TKI, further demonstrated a significantly improved OS in ARCHER 1050 trial comprising a cohort of patients without brain metastasis^22,23^. Recently, Jung et al. reported a different CNS efficacy between first- and second-generation TKIs in a real-world cohort of *EGFR*-mutant patients in which a lower CNS progression rate was observed in afatinib-treated patients^24^. However, given the higher treatment-related toxicity of afatinib than gefitinib/erlotinib, the frequent requirement of dose modification also raises the concern of suboptimal brain concentration secondary to the penetrant hurdle of blood brain barrier. As a result, Tan et al. has demonstrated that, in *EGFR*-mutant patients with brain metastasis, dose reduction of afatinib was associated with a reduced PFS^25^. Taken together, more data are still required to understand the joint effect regarding brain metastasis and generation of EGFR-TKI on the outcome of *EGFR*-mutant patients.

The two subtypes of common *EGFR* mutation, the L858R in exon 21 and the deletion in exon 19, are also closely implicated in patients’ outcome. Previous studies have revealed that *EGFR* L858R mutants usually harbour more co-occurring mutations, both within and beyond the *EGFR* gene, than *EGFR* 19 deletion mutants^26,27^. As a result, studies which involved gefitinib/erlotinib or osimertinib treatment for *EGFR*-mutant NSCLC frequently showed lower therapeutic efficacies in L858R patients compared to 19 deletion ones^28,29^. Nevertheless, this genotype-dependent treatment efficacy seems less evident in afatinib-treated patients^22,30^. In addition, the differences in terms of OS and secondary T790M mutation rate have also been reported between the two common *EGFR* genotypes^31,32^. Overall, how these clinical, molecular features and therapeutic parameter jointly influence the outcome of *EGFR*-mutant NSCLC requires the analysis from a large patient sample.

In this study, we analyzed a large, real-world cohort of metastatic *EGFR*-mutant NSCLC patients who received front-line treatment of gefitinib/erlotinib or afatinib. The survival outcome and secondary T790M mutation rate in relation to brain metastasis, *EGFR* subtypes and generation of EGFR-TKI administration were reported.

## Methods

### Patients and treatment

Patients diagnosed metastatic NSCLC with *EGFR*-sensitizing mutation, the exon 19 deletion or the exon 21 L858R mutation, who received first-line treatment of gefitinib, erlotinib or afatinib between January 2013 and December 2019 were included in present study. Patient received the treatment of gefitinib 250 mg, erlotinib 150 mg or afatinib 40 mg as the starting dose and those who received the treatment less than 3 weeks were excluded from the analysis. Acquired *EGFR* T790M mutation was tested by a therascreen® EGFR RGQ PCR kit (QIAGEN, Valencia, USA) for patients who had available tumor tissues and/or by plasma cell-free DNA (liquid biopsy) using a RainDrop™ Digital PCR System (RainDance Technologies, Boston, USA). The progression-free survival (PFS) was defined as the interval between the date of starting EGFR-TKI and the date of radiologically or clinically determined progression or death. The treatment response, including complete response (CR), partial response (PR), stable disease, and progressive disease, was evaluated according to the Response Evaluation Criteria in Solid Tumors (version 1.1). The study used data from the Chang Gung Research Database and the Ethics Committee of Chang Gung Memorial Hospital approved the study protocol and the waiver of informed consent form (No. 201801967B0).

### Statistical analysis

The Mann–Whitney test was used to determine the statistical significance of continuous variables between the two groups and Chi-squared test was used for evaluating the categorical variables. The Kaplan–Meier survival curve was analysed using the R package *survival*, and the hazard ratio (HR) was analysed using the Cox regression model. The patterns of disease progression, a CNS progression alone or a systemic progression, were treated as competing risk events of which the cumulative incidence functions were calculated^33^. The modified Cox regression model for the subdistribution hazard of the cumulative incidence function was applied to calculate the disease progression hazard from a given pattern in the presence of competing events by using the R package *cmprsk*^34^. All the reported p values were two sided, and a *p* < 0.05 was considered statistically significant. Data were also analyzed using SPSS (version 10.1; SPSS, Chicago, IL, USA).

### Ethics statement

The study was performed in accordance with the ethical standards of the 1964 Declaration of Helsinki. The Ethics Committee of Chang Gung Memorial Hospital approved the study (No. 201801967B0) and granted permission for access to the Chang Gung Research Database and the IRB approved the waiver of the informed consent form.

## Results

### Baseline patient characteristics by brain metastasis status

A total of 553 patients were included for analysis, in which 211 (38.2%) patients presented baseline brain metastasis and 342 (61.8%) patients were absent of baseline brain metastasis (Table 1). The clinical features including age, sex, smoking history, histology and *EGFR* mutation subtype were similar between the two groups. However, patients with baseline brain metastasis had a significantly higher frequency of co-occurring liver metastasis (17.1% vs. 9.6%, p = 0.012) and were significantly more likely to have an ECOG performance status ≥ 2 (21.8% vs. 14.0%, p = 0.020) compared to those without baseline brain metastasis. Notably, physician were more likely to prescribe first-generation EGFR-TKI (53.6% vs. 38.0%, p < 0.001) for patients presenting brain metastasis in clinical practice compared to those absent of brain metastasis (Table 1). The median follow-up duration was 33.6 months and 272 (49.2%) events of death were observed at the time of analysis.

**Table 1:** Clinical characteristics by brain metastasis.

	Total (%), N = 553	With brain metastasis, N = 211	Without brain metastasis, N = 342	p value
Age				
Mean ± SD	66.8 ± 11.9	66.0 ± 11.5	67.2 ± 12.1	0.220
≥ 65 years old	312 (56.4)	117 (55.5)	195 (57.0)	0.725
ECOG PS				
0–1	459 (83.0)	165 (78.2)	294 (86.0)	0.020
≥ 2	94 (17.0)	46 (21.8)	48 (14.0)	
Sex				
Male	212 (38.3)	75 (35.5)	137 (40.1)	0.322
Female	341 (61.7)	136 (64.5)	205 (59.9)	
Smoking history				
Current/ex-smoker	127 (23.0)	50 (23.7)	77 (22.5)	0.756
Never smoker	426 (77.0)	161 (76.3)	265 (77.5)	
Histology				
Adenocarcinoma	545 (98.6)	209 (99.1)	336 (98.2)	0.717
Others	8 (1.4)	2 (0.9)	6 (1.8)	
*EGFR* mutation				
L858R	296 (53.5)	117 (55.5)	179 (52.3)	0.484
19 deletion	257 (46.5)	94 (44.5)	163 (47.7)	
EGFR-TKI				
Gefitinib/erlotinib	243 (43.9)	113 (53.6)	130 (38.0)	< 0.001
Afatinib	310 (56.1)	98 (46.4)	212 (62.0)	
Liver metastasis				
Presence	69 (12.5)	36 (17.1)	33 (9.6)	0.012
Absence	484 (87.5)	175 (82.9)	309 (90.4)	

### Cox regression analysis of PFS

**Figure 1:**
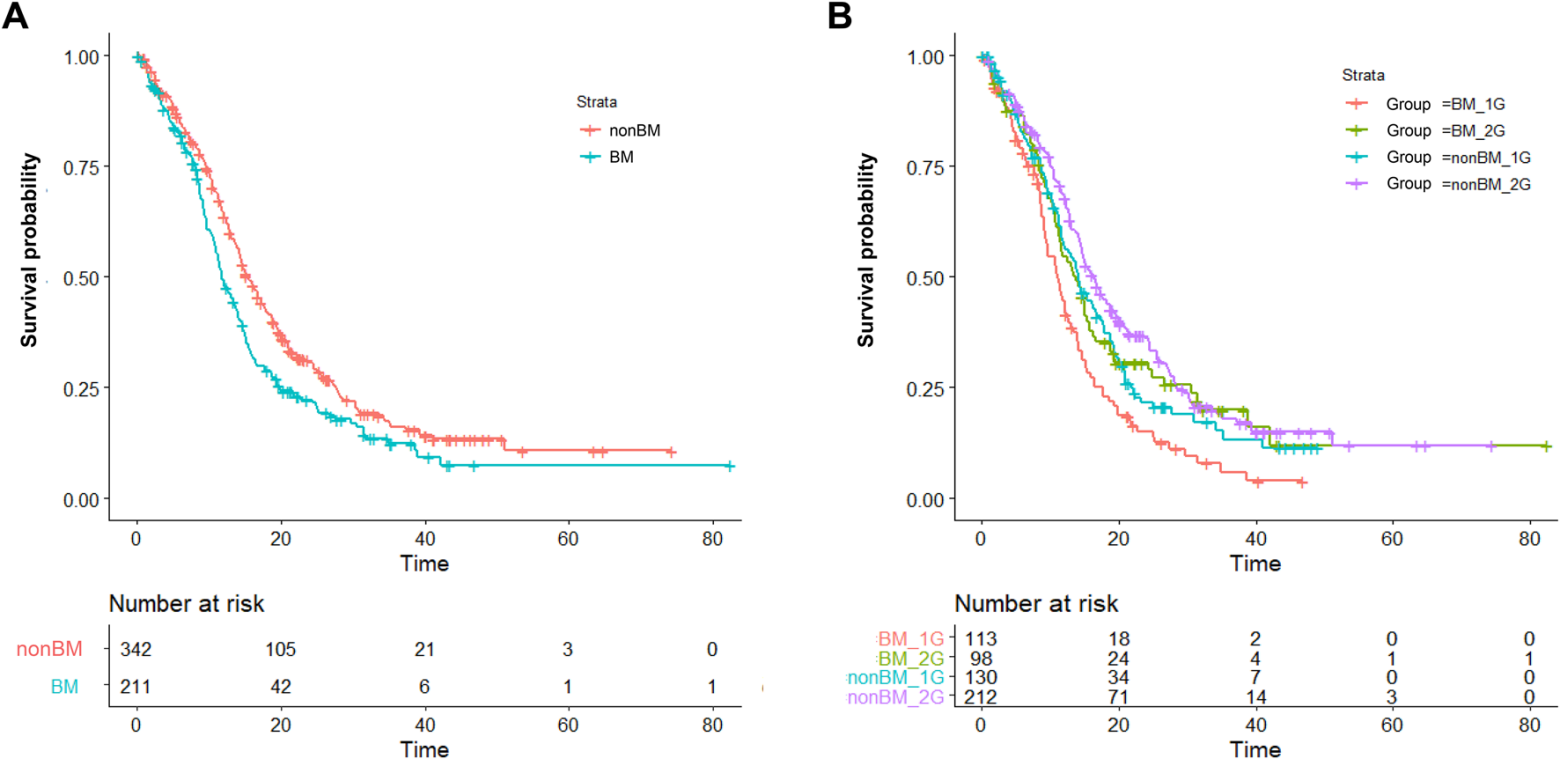
The PFS between patients grouped by (**A**) with or without BM (**B**) with or without BM and first- or second-generation EGFR-TKI treatment. *BM* brain metastasis, *1G* first-generation EGFR-TKI, *2G* second-generation EGFR-TKI.

Univariate Cox regression analyses demonstrated that brain metastasis (HR 1.34 [95% CI 1.10–1.64]; p = 0.003), ECOG PS 0–1 (HR 0.58 [95% CI 0.45–0.74]; p < 0.001), second-generation EGFR-TKI afatinib treatment (HR 0.71 [95% CI 0.59–0.86]; p < 0.001) and liver metastasis (HR 1.56 [95% CI 1.19–2.04]; p = 0.001, Table 2) had significant impact on PFS. Multivariate regression adjustment exhibited that ECOG PS 0–1 (HR 0.71 [95% CI 0.54–0.93]; p = 0.014), second-generation EGFR-TKI afatinib treatment (HR 0.81 [95% CI 0.66–0.99]; p = 0.045) and liver metastasis (HR 1.31 [95% CI 1.00–1.77]; p = 0.047, Table 2) were independent predictors of PFS. In addition, a trend of negative impact on PFS remained observed for brain metastasis (HR 1.20 [95% CI 0.99–1.48]; p = 0.053, Table 2) where patients with brain metastasis showed a shorter median PFS (11.8 vs. 15.4 months; log-rank test p = 0.003), a lower 24-month PFS rate (22.3% [95% CI 17.0% to 29.3%] vs. 30.7% [95% CI 25.9% to 36.5%], Fig. 1A) compared to those without.

**Table 2:** Cox regression analysis of progression-free survival.

Variable	Univariate analysis			Multivariate analysis		
	HR	95% CI	p value	HR	95% CI	p value
Age ≥ 65	1.13	0.93–1.37	0.217	–	–	–
ECOG 0, 1	0.58	0.45–0.74	< 0.001	0.71	0.54–0.93	0.014
Male	1.09	0.90–1.33	0.369	–	–	–
Current/ex-smoker	1.18	0.94–1.48	0.146	–	–	–
*EGFR* L858R	1.21	0.99–1.46	0.052	–	–	–
Afatinib treatment	0.71	0.59–0.86	< 0.001	0.81	0.66–0.99	0.045
Brain metastasis	1.34	1.10–1.64	0.003	1.20	0.99–1.48	0.053
Liver metastasis	1.56	1.19–2.04	0.001	1.31	1.00–1.77	0.047

### PFS and pattern of progression by brain metastasis status and generation of EGFR-TKI

**Figure 2:**
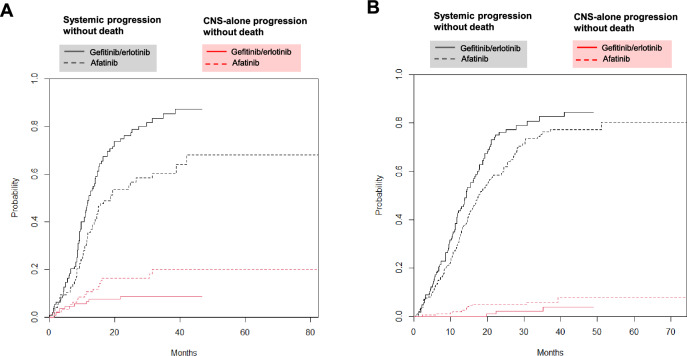
Cumulative incidence of systemic progression without death (black) and CNS- alone progression without death (red) between first-generation EGFR-TKI gefitinib/erlotinib (solid line) and second-generation EGFR-TKI afatinib (broken line) in patients (**A**) with brain metastasis and (**B**) without brain metastasis.

We subsequently analysed the patient groups stratified by brain metastasis status and generation of EGFR-TKI treatment. Patients who received second-generation EGFR-TKI afatinib, irrespective of brain metastasis status, were generally younger, with better ECOG PS and had more *EGFR* 19 deletion genotype, compared to patients who received first-generation EGFR-TKI gefitinib/erlotinib (Table 3). Putting together, afatinib-treated patients without brain metastasis demonstrated a significantly longer PFS (16.3 months) compared to afatinib-treated patients with brain metastasis (13.7 months) and to gefitinib/erlotinib-treated patients with (11.1 months) or without brain metastasis (14.2 months; log-rank test p < 0.001, Fig. 1B). As to the pattern of disease progression: In brain metastasis group, afatinib-treated patients demonstrated a significantly lower rate of systemic progression (adjusted cause-specific HR, 0.54; 95% CI 0.38–0.77; p < 0.001) but a trend of higher CNS-alone progression (adjusted cause-specific HR, 2.28; 95% CI 0.88–5.87; p = 0.086, Fig. 2A), compared to gefitinib/erlotinib-treated patients. In non-brain metastasis group, similar systemic progression (adjusted cause-specific HR, 0.87; 95% CI 0.65–1.18; p = 0.370) and CNS-alone progression (adjusted cause-specific HR, 3.02; 95% CI 0.71–12.9; p = 0.140, Fig. 2B) were observed between afatinib- and gefitinib/erlotinib-treated patients.

**Table 3:** Clinical characteristics by brain metastasis and generation of EGFR-TKI.

Number (%)	With brain metastasis		p-value	Without brain metastasis		p-value
	Gefitinib/erlotinib, N = 113	Afatinib, N = 98		Gefitinib/erlotinib, N = 130	Afatinib, N = 212	
Age						
Mean ± SD	68.6 ± 11.8	62.9 ± 10.3	< 0.001	72.9 ± 12.0	63.7 ± 10.7	< 0.001
≥ 65 years old	74 (65.5)	43 (43.9)	0.003	101 (77.7)	94 (44.3)	< 0.001
ECOG PS						
0–1	76 (67.3)	89 (90.8)		100 (76.9)	194 (91.5)	
≥ 2	37 (32.7)	9 (9.2)	< 0.001	30 (23.1)	18 (8.5)	< 0.001
Sex						
Male	40 (35.4)	35 (35.7)		45 (34.6)	92 (43.4)	
Female	73 (64.6)	63 (64.3)	1.000	85 (65.4)	120 (56.6)	0.135
Smoking history						
Current/ex-smoker	27 (23.9)	23 (23.5)		34 (26.2)	43 (20.3)	
Never smoker	86 (76.1)	75 (76.5)	1.000	96 (73.8)	169 (79.7)	0.259
Histology						
Adenocarcinoma	112 (99.1)	97 (99.0)		128 (98.5)	208 (98.1)	
Others	1 (0.9)	1 (1.0)	1.000	2 (1.5)	4 (1.9)	1.000
*EGFR* mutation						
L858R	71 (62.8)	46 (46.9)		80 (61.5)	99 (46.7)	
19 deletion	42 (37.2)	52 (53.1)	0.029	50 (38.5)	113 (53.3)	0.011
Liver metastasis						
Presence	19 (16.8)	18 (18.4)		13 (10.0)	20 (9.4)	
Absence	95 (83.2)	80 (81.6)	0.775	117 (90.0)	192 (90.6)	1.000

### OS by brain metastasis and Cox regression analysis

**Figure 3:**
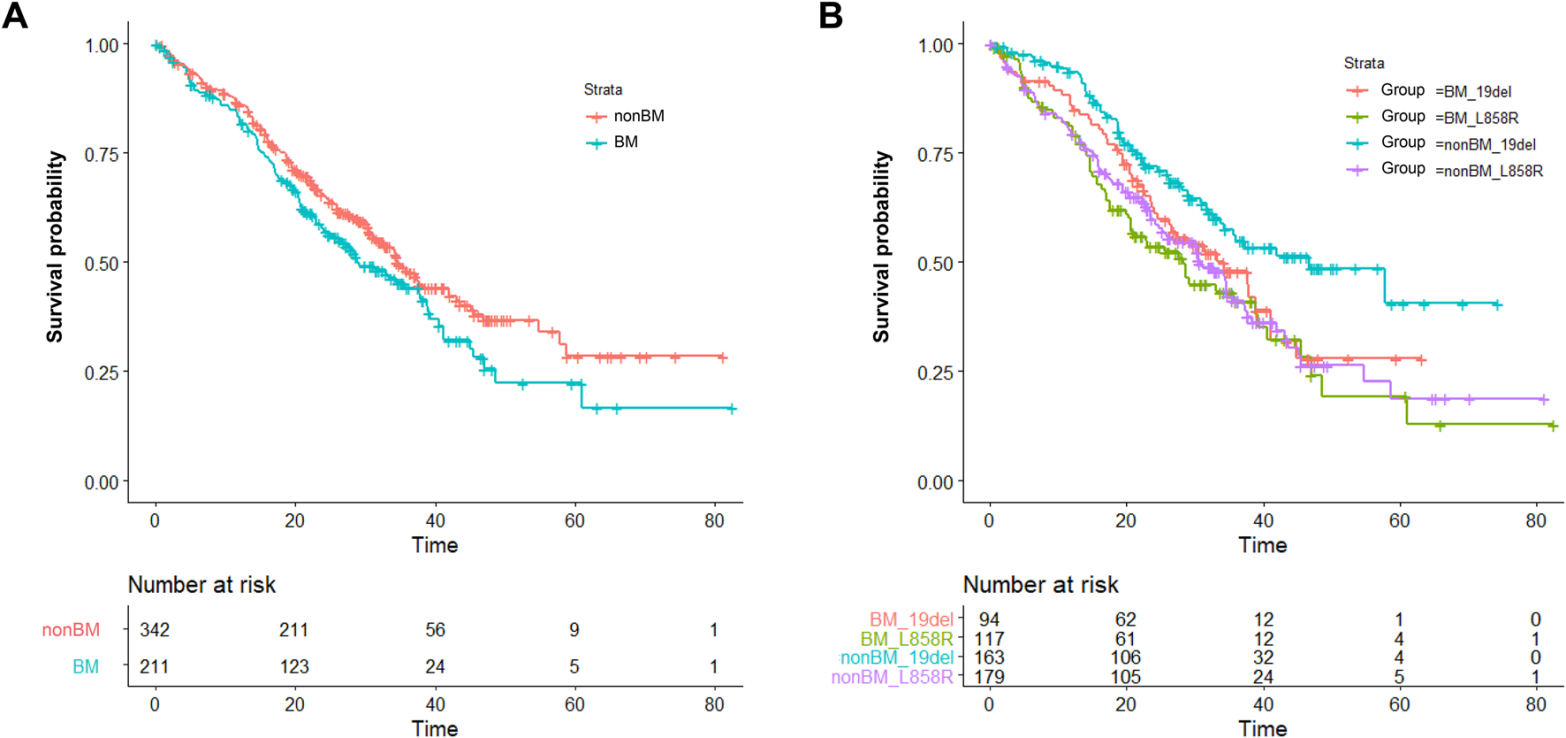
The OS between patients grouped by (**A**) with or without BM (**B**) with or without BM and *EGFR* mutation subtype. *BM* brain metastasis, *19del EGFR* 19 deletion mutation, *L858R EGFR* L858R mutation.

Patients with brain metastasis, compared to those without, had a numerically shorter OS (28.9 vs. 34.7 months; log-rank test p = 0.060, Fig. 3A) and a trend of increased risk of death (HR 1.26 [95% CI 0.99–1.60]; p = 0.063, Table 4). Univariate Cox regression demonstrated that older patients of age ≥ 65 (HR 1.33 [95% CI 1.05–1.69]; p = 0.019), ECOG PS 0–1 (HR 0.38 [95% CI 0.29–0.50]; p < 0.001), *EGFR* L858R mutation (HR 1.32 [95% CI 1.01–1.72]; p = 0.044), second-generation EGFR-TKI afatinib treatment (HR 0.69 [95% CI 0.54–0.87]; p = 0.002) and liver metastasis (HR 1.47 [95% CI 1.06–2.04]; p = 0.022, Table 4) also had significant impact on OS. Multivariate adjustment demonstrated that ECOG PS 0–1 (HR 0.41 [95% CI 0.31–0.56]; p < 0.001) and *EGFR* L858R mutation (HR 1.46 [95% CI 1.13–1.88]; p = 0.003, Table 4) were the independent factors predictive of OS. We later analysed the OS stratified by brain metastasis status and *EGFR* mutation subtype. Patients who had *EGFR* 19 deletion mutation and without brain metastasis exhibited a significantly longer OS (46.8 months) compared to *EGFR* 19 deletion patients with brain metastasis (34.3 months), *EGFR* L858R mutation with (28.3 months) and without (30.7 months; log-rank test p = 0.002, Fig. 3B) brain metastasis.

**Table 4:** Cox regression analysis of overall survival.

Variable	Univariate analysis			Multivariate analysis		
	HR	95% CI	p value	HR	95% CI	p value
Age ≥ 65	1.33	1.05–1.69	0.019	1.19	0.92–1.54	0.184
ECOG 0, 1	0.38	0.29–0.50	< 0.001	0.41	0.31–0.56	< 0.001
Male	1.07	0.84–1.36	0.576	–	–	–
Current/ex-smoker	1.16	0.88–1.52	0.290	–	–	–
*EGFR* L858R	1.32	1.01–1.72	0.044	1.46	1.13–1.88	0.003
Afatinib treatment	0.69	0.54– 0.87	0.002	0.98	0.75–1.28	0.893
Brain metastasis	1.26	0.99–1.60	0.063	1.10	0.86–1.42	0.431
Liver metastasis	1.47	1.06–2.04	0.022	1.36	0.97–1.92	0.072

### Brain metastasis status as a predictor of secondary T790M development

A total of 182 (32.9%) patients underwent an *EGFR* T790M mutation assay by tissue and/or liquid biopsy upon disease progression. A positive T790M status was determined in 101 (55.5%) patients and the T790M rate was similar between tissue (67/127, 52.7%) and liquid (34/67, 51.5%; Chi-squared p = 0.908) biopsy. Clinical factors including brain metastasis status, *EGFR* mutation subtype, generation of EGFR-TKI treatment and PFS longer than 12 months were all observed to be associated with the T790M positive rate by univariate logistic regression **(**Table 5). Multivariate analysis demonstrated that brain metastasis (OR 2.02 [95% CI 1.02–4.08]; p = 0.040), second-generation EGFR-TKI afatinib treatment (OR 0.26 [95% CI 0.12–0.50]; p < 0.001) and PFS longer than 12 months (OR 3.10 [95% CI 1.59–6.30]; p = 0.001, Table 5) were independent predictors to the secondary T790M rate. The *EGFR* L858R mutation (OR 0.55 [95% CI 0.28–1.05]; p = 0.070) was associated with a trend of lower T790M positivity after multivariate adjustment.

**Table 5:** Analysis of factors predictive of T790M positive rate.

Variables	Univariate analysis		Mutivariate analysis	
	Odd ratio (95% CI)	p-value	Odd ratio (95% CI)	p-value
Age ≥ 65	0.91 (0.51–1.63)	0.748	–	–
Male	0.86 (0.48–1.54)	0.606	–	–
ECOG PS 0–1	0.68 (0.27–1.60)	0.383	–	–
Current/ex-smoker	0.84 (0.44–1.60)	0.593	–	–
*EGFR* L858R	0.59 (0.33–1.06)	0.081	0.55 (0.28–1.05)	0.07
Brain metastasis	2.07 (1.12–3.87)	0.021	2.02 (1.02–4.08)	0.04
Liver metastasis	1.49 (0.63–3.71)	0.370	–	–
Afatinib treatment	0.33 (0.17–0.60)	< 0.001	0.26 (0.12–0.50)	< 0.001
PFS ≥ 12 months	2.00 (1.11–3.63)	0.021	3.10 (1.59–6.30)	0.001

### Determination of T790M rate by brain metastasis status-based subgroups

**Figure 4:**
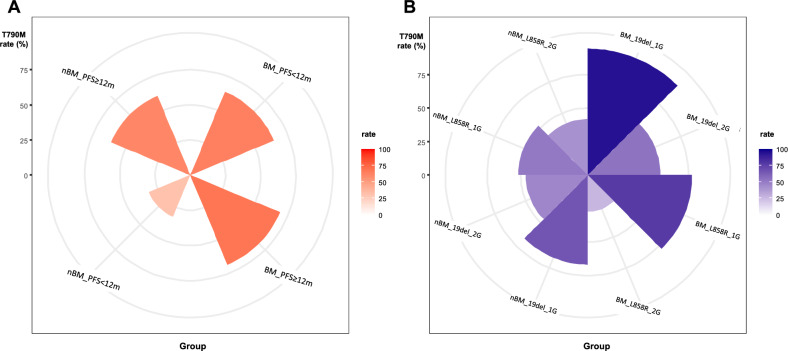
The secondary T790M rate between patients grouped by (**A**) with or without BM and PFS ≥ or < 12 months (**B**) with or without BM, EGFR mutation subtype and generation of EGFR-TKI treatment. *BM* brain metastasis, *19del EGFR* 19 deletion mutation, *L858R EGFR* L858R mutation, *1G* first-generation EGFR-TKI, *2G* second-generation EGFR-TKI.

For patient groups stratified by brain metastasis status and length of PFS: The group without brain metastasis and PFS less than 12 months demonstrated a lowest secondary T790M rate compared to the others (31.9%; Chi-squared p = 0.002, Fig. 4A). For patient groups stratified by brain metastasis status, *EGFR* mutation subtype and generation of EGFR-TKI treatment: The group with brain metastasis and *EGFR* 19 deletion mutation receiving gefitinib/erlotinib demonstrated a highest T790M rate (94.4%) and the group with brain metastasis and L858R mutation receiving afatinib exhibited a lowest T790M rate (27.3%; Chi-squared p < 0.001, Fig. 4B), among others.

## Discussion

The present study demonstrated that the presence of brain metastasis in *EGFR*-mutant NSCLC patients had clinical impact on the efficacy of front-line EGFR-TKI treatment, particularly for the first-generation one; whereas the impact toward OS was not significant. The generation of EGFR-TKI administration also jointly influenced the treatment efficacy. The *EGFR* mutation subtype mainly has an impact on the patients’ overall survival.

We have previously reported that the generation EGFR-TKI used was associated with therapeutic efficacy in *EGFR*-mutant NSCLC patients, in which gefitinib/erlotinib-treated patients presented a shorter PFS compared to afatinib-treated ones^11^. In this analysis, we demonstrated that brain metastasis further impacted the therapeutic efficacy of gefitinib/erlotinib whereas this impact on the efficacy of afatinib treatment seemed to be less significant. Thus, this finding indicated that afatinib may be a better treatment option than gefitinib/erlotinib in *EGFR*-positive patients with brain metastasis. Recently, a similar finding has also been reported by Jung et al. that patient who received afatinib treatment experienced a significantly longer CNS-PFS than those who received gefitinib or erlotinib^24^. These seemingly higher efficacies of afatinib in brain metastasis cohort may be partly explained by the previous studies which analyzed first- and second-generation EGFR-TKI concentrations in human cerebralspinal fluid^35,36^ where afatinib treatment was likely associated with a larger margin of differential between the measured drug concentration in cerebralspinal fluid and the reported data of in vitro IC_50_ against NSCLC cell lines with* EGFR*-sensitizing mutation^37^.

Nevertheless, the higher toxicity profile of afatinib treatment and thereby the related dose reduction or interruption also has been linked to a reduced efficacy previously^25^. Whether this can be associated with more CNS-alone progression, as a result of reduced serum concentration in the face of blood–brain barrier, was largely unknown. In the present study, a non-significantly higher hazard of CNS-alone progression was observed in afatinib-treated patients of whom 56.3% has undergone dose reduction during the treatment. This finding suggested that a specific attention may be paid to CNS-alone progression for afatinib-treated patients especially when they underwent dose modification even tumors were well-controlled in primary site or other non-CNS metastatic sites. Recently, dacomitinib has demonstrated excellent intracranial response with both standard and reduced dose scheme in a real-world cohort of *EGFR*-mutant patients^38^. However, whether dose reduction has an impact on the duration of CNS response and progression requires further investigation. Despite the status of brain metastasis implicated in the treatment efficacy of EGFR-TKI, it did not seem to impose significant impact on the patients’ overall survival in this analysis. This finding suggested that the efficacy of post-progression treatment was minimally compromised in the patients with brain metastasis likely because their disease burden in brain was successfully controlled. Following the line, the fact that approximately 60% of the patients with brain metastasis in the present study received EGFR-TKI alone without upfront brain radiotherapy might also support the mindset of deferring radiotherapy for selected patients without undermining their survival^39^.

Regarding to the prognostic impact of *EGFR* L858R and 19 deletion mutations, the issue remains pretty much unsettled as the findings from previous studies have been quite controversial^40^. In this study, we demonstrated that subtype of *EGFR* mutation had significant impact on OS in which patients with *EGFR* L858R mutation had less favorable OS compared to those with *EGFR* 19 deletion mutation. The explanation to this finding can likely be multi-faceted. Previous studies have revealed that NSCLC with L858R mutation tended to possess a higher number of co-occurring genomic alterations than NSCLC with 19 deletion mutation^26,27^. This increases the clonal complexity of the tumors and naturally places them in a higher likelihood to avert targeted therapies^28,29^. Other studies reported elsewhere further observed a higher frequency of primary *EGFR* T790M mutation in L858R patients compared to 19 deletion ones and thus associated with the primary resistance to first or second-generation EGFR-TKI treatments^41,42^. In contrary, the frequency of secondary *EGFR* T790M mutation was reported to be lower in L858R patients than in 19 deletion ones, both in previous studies and the present one^43,44^. This usually rendered patients whose disease progressed from first or second-generation EGFR-TKIs unavailable to the approved treatment of third-generation T790M-active drugs and thereby leading to a less favorable survival outcome. However, more data analysis from clinical trial and real world will be required to determine the true prognostic impact between the two common sensitizing mutations.

Along this line, identification of secondary *EGFR* T790M mutation in patients who progress from geftinib/erlotinib or afatinib treatment is of paramount importance. However, this is usually limited by unavailable/insufficient tumor tissue, unqualified nucleic acid, additional testing cost and, not infrequently, rapid disease progression^45,46^. In this analysis, several parameters were identified to be associated with secondary T790M rate including status of brain metastasis, PFS length of front-line EGFR-TKI treatment, generation of EGFR-TKI used and *EGFR* mutation subtype. We further observed differential secondary T790M rates in patients defined by two sets of brain metastasis-based parameters. The T790M rate was approximately 60% ~ 70% in patients with baseline brain metastasis irrespective of the PFS length of EGFR-TKI treatment (≥ or < 12 months). However, further dissection toward these patients revealed that *EGFR* 19 deletion cohort treated by gefitinib/erlotinib and *EGFR* L858R cohort treated by afatinib had the highest and the lowest T790M rates (94.4% vs. 27.3%), respectively. In patient without baseline brain metastasis, the T790M rates were similar (42% ~ 67%) among the four patient cohorts defined by *EGFR* mutation subtype and generation of EGFR-TKI used. However, the PFS length was significantly associated with T790M rate in these patients, where those who experienced a PFS < 12 months had a lower T790M rate compared to those who experienced a PFS ≥ 12 months (31.9% vs. 60.9%).

The limitation of this study, firstly, lies in its retrospective nature. Secondly, the front-line osimertinib has currently become the recommended standard of care for patients of advanced *EGFR*-mutant NSCLC, with the advantage for treatment or prevention of brain metastasis. However, the findings of the study would be important in places where osimertinib is not available to patients due to various reasons. Thirdly, clinical characteristics were imbalanced between patients with or without brain metastasis in the study. However, these biases reasonably reflected the different condition intrinsically existed between the two groups of patients and the exact impact against survival outcome was also clarified by statistical adjustment with regression-based method. Overall, this analysis demonstrated that status of brain metastasis and generation of EGFR-TKI have joint impact on the PFS of TKI treatment while the OS is mainly determined by *EGFR* mutation subtype. The differential secondary T790M rate may be further predicted by these three parameters. Additional prospective studies are warranted to validate these findings.

## Data Availability

The datasets generated and/or analysed in the current study are available from the corresponding author on reasonable request.
